# Identification of misexpressed genetic elements in hybrids between Drosophila-related species

**DOI:** 10.1038/srep40618

**Published:** 2017-01-16

**Authors:** Hélène Lopez-Maestre, Elias A. G. Carnelossi, Vincent Lacroix, Nelly Burlet, Bruno Mugat, Séverine Chambeyron, Claudia M. A. Carareto, Cristina Vieira

**Affiliations:** 1 Laboratoire de Biométrie et Biologie Evolutive, UMR CNRS 5558, Université Lyon 1, Université de Lyon, Villeurbanne, France; 2 ERABLE-team, INRIA Grenoble Rhône-Alpes, France; 3 Department of Biology, UNESP–São Paulo State University, São José do Rio Preto, São Paulo, Brazil; 4 Institut de Génétique Humaine, Centre National de la Recherche Scientifique, UPR1142, Montpellier, France

## Abstract

Crosses between close species can lead to genomic disorders, often considered to be the cause of hybrid incompatibility, one of the initial steps in the speciation process. How these incompatibilities are established and what are their causes remain unclear. To understand the initiation of hybrid incompatibility, we performed reciprocal crosses between two species of Drosophila (*D. mojavensis* and *D. arizonae*) that diverged less than 1 Mya. We performed a genome-wide transcriptomic analysis on ovaries from parental lines and on hybrids from reciprocal crosses. Using an innovative procedure of co-assembling transcriptomes, we show that parental lines differ in the expression of their genes and transposable elements. Reciprocal hybrids presented specific gene categories and few transposable element families misexpressed relative to the parental lines. Because TEs are mainly silenced by piwi-interacting RNAs (piRNAs), we hypothesize that in hybrids the deregulation of specific TE families is due to the absence of such small RNAs. Small RNA sequencing confirmed our hypothesis and we therefore propose that TEs can indeed be major players of genome differentiation and be implicated in the first steps of genomic incompatibilities through small RNA regulation.

Interspecific hybridization can be considered as a stress condition with multiple consequences for the hybrid genome. It may cause chromosomal rearrangements, inversions, deletions, changes in gene expression, changes in DNA methylation, among other effects^1^,^2^. Global activation of transposable elements (TEs), which induces profound changes in the hybrid genome, has also been described. Such changes generate new phenotypes and the formation of reproductively isolated populations because the accumulation of structural and functional genomic changes acts as a pressure leading to speciation^3^,^4^,^5^. For example, hybrid *Helianthus*, derived from crosses of the same parental species with other hybrids, have 50% more nuclear DNA than the parental species, mainly due to bursts of transposition^6^. Interspecific hybrids of kangaroos from the Macropodidae family also showed variation in amplification of satellite repeats and kerV-1 element, changes in chromatin structure and rearrangements of whole chromosome arms^7^, which demonstrates that during hybridization, increased transposition is observed, inducing significant changes in karyotype^3^,^8^.

In *Drosophila,* studies of intraspecific crosses revealed asymmetric sterility of the offspring. This phenomenon was named hybrid dysgenesis and was first described in the 1960s in *D. melanogaster* with the I/R system^9^ and then the P/M system^10^. Hybrid dysgenesis corresponds to aberrant phenotypic traits observed in the F1 of crosses between particular strains or natural populations and was proposed as a possible driver of speciation^1^,^11^. Hybrid dysgenesis was attributed to differences in TE contents between parental lines. We now know that TEs are major components of the genome architecture because they may encompass a large fraction of the genome size and may trigger recombination. However, we also know that most of the TEs in the genomes are inactive. The last decade shed light on TE epigenetic control. In *Drosophila*, most TEs are post-transcriptionally silenced via a particular class of small RNAs, called piRNAs (piwi-interacting RNAs)^12^,^13^,^14^. Subsequently, transcriptional silencing is also caused by chemical histone modifications, which change the chromatin structure^15^,^16^. When the efficiency of the effectors of these pathways is no longer maintained, TEs may transpose into genomes, which leads to significant decreases in fitness, including lethality^17^,^18^,^19^. Due to the recent development of our knowledge in epigenetics, we know that hybrid dysgenesis is caused by differences in the piRNA contents between the parental lines. When two strains display different TE contents, and therefore different associated piRNA contents, a cross between a male with an active TE family and a female devoid of the corresponding piRNAs leads to a major increase in TE expression, disrupting the genome stability, which could result in sterility or lethality^20^,^21^. Hybrid dysgenesis also occurs in *D. virilis* and is due to the death of germ cells during embryogenesis probably related to the initiation of transcription of several retrotransposons^22^. In artificially interspecific hybrids between *D. melanogaster* and *D. simulans*, TEs are derepressed due to adaptive divergence in the piRNA genes of both species rather than differences in TE contents^23^. Other studies with crosses between *D. buzzatti* and *D. koepferae* have shown that 70% of the genomic rearrangements observed in hybrids was due to TE insertions^24^.

To understand the first steps in hybrid incompatibility, we propose the use of related species that diverged recently (less than 1 Mya). *D. arizonae* and *D. mojavensis* are endemic species of the arid southwestern United States and Mexico (Fig. 1A). *D. arizonae* occurs in the cape region in Baja California, southeastern Arizona, southeastern New Mexico, the southeastern Sonoran Desert, eastern Mexico and Guatemala. *D. mojavensis* occurs in the Mojave and Sonoran Deserts, southern California and Baja California (USA) and along the west coast of Sonora and Sinaloa (Mexico), where it is sympatric with *D. arizonae*^25^,^26^,^27^. The two species diverged recently (between 0.6 and 1 Mya)^28^,^29^,^30^ and the degree of pre-zygotic isolation between them is strong, but it is incomplete and variable, depending on the geographic origin of the populations. The pre-zygotic isolation is higher between the sympatric than the allopatric populations^25^,^31^,^32^. Hybridization between the two species does not occur in nature or is extremely rare^25^,^27^, but in the laboratory, crosses between *D. mojavensis* and *D. arizonae* are possible and present variation in the degree of sterility of the males^33^,^34^. A genome-wide expression study of these two species investigated transcriptional changes in relation to pre-zygotic mechanisms of isolation^35^, and to our knowledge, no data are available for transcriptome changes that might influence postzygotic mechanisms. We chose to cross two allopatric strains for which we can obtain hybrids in the laboratory and analyzed the transcriptomes from the female ovaries of both parental and reciprocal hybrids (Fig. 1B).

We show that reciprocal hybrids presented average levels of gene expression compared to the parental lines, with some specific gene categories being misexpressed such as genes related to embryo development. As for TEs, we identified few families that were highly expressed in hybrid crosses relative to the parental lines. Because TEs are mainly silenced by small RNAs from the piwi small RNA class (piRNA), we show that in hybrids the deregulation of specific TE families is due to the absence of such small RNAs. Indeed, small RNA sequencing confirms our hypothesis and we therefore propose that TEs can indeed be major players in genome differentiation and be involved in the first steps of genomic incompatibilities through small RNA regulation.

## Results

### Co-assembling-Quantification-Genes and TE identification

**Figure 1:**
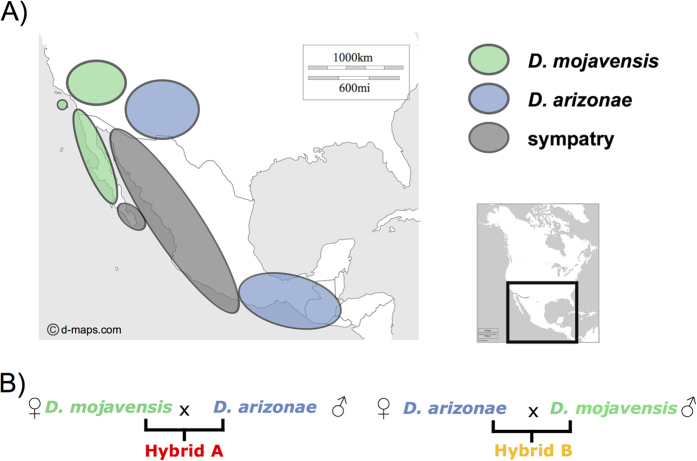
(**A**) Geographic distribution of *D. mojavensis* and *D. arizonae.* The two species occupy the south USA and Mexico with strains in sympatry and allopatry. The two strains used in this study come from allopatric regions (http://www.d-maps.com/carte.php?num_car=1404&lang=en). (**B**) Crosses between *D. mojavensis* and *D. arizonae.* Reciprocal crosses were performed between the species with allopatric strains (see Materials and Methods). We named crosses made with *D. mojavensis* females hybrid A and crosses made with *D. arizonae* females hybrid B.

We sequenced the ovarian transcriptomes of two parental allopatric strains (*D. mojavensis* and *D. arizonae*) (Fig. 1A) and of reciprocal hybrid crosses (named hereafter as crosses Hybrid A and B, see Fig. 1B). We obtained a total of 700 million paired-end reads, corresponding on average to 100 to 130 million reads for each of the parental and hybrid libraries (3 biological replicates for each condition). To produce a reference transcriptome, we use all quality reads from parental species and hybrids (co-assembly). Our choice to co-assemble all reads was motivated by the following reasons: (1) no reference genome was available for *D. arizonae*; hence, mapping all reads to the *D. mojavensis* genome might have biased the results towards the genes of *D. mojavensis*; and (2) assembling each dataset separately results in a poor resolution for genes that are moderately or lowly expressed.

The assembler we used, Trinity, assemble sequences that can correspond to the same “component”, i.e. sequences that can come from the same gene (see Fig. S1). To control the performance of the co-assembly, we verified that the number of components obtained by Trinity was higher when co-assembling (21,888) than with a separate assembly for each species (~15,000). We also verified that the cumulated length of the longest sequences per gene was higher for the co-assembly (24 Gb) than for the individual assemblies of each dataset (~19 Gb) (SM Table 1). Moreover, from the 21,888 components obtained in the co-assembly, 14,957 have one associated component (see Material and Methods) in at least one of the single assemblies. The remaining 6,932 components were lost when using single species assemblies, showing the benefit of the co-assembly procedure.

One risk of co-assembling is the increased possibility of generating chimeric sequences. To estimate their number, we searched for assembled sequences that do not align on the reference genome of *D. mojavensis* and found that the number of such sequences was similar for the different assembly procedures, namely on average 5% of all the sequences (Table S1).

When mapping the sequence reads back to the assemblies, we obtained a high back-mapping rate for the co-assembly (98.5%), which is also slightly higher than the back-mapping rates obtained for each single assembly (Table S1). We further verified that the nucleotide divergence between the two species did not reduce the efficiency of the assembly, with genes that are more divergent being less well assembled. We used the assemblies of *D. mojavensis* and *D. arizonae*, identified the orthologous genes, and calculated the nucleotide divergence. The average divergence rate between parental species is less than 2%, and less than 5% for 95% of the sequences. These results suggest that the nucleotide divergence between the species is sufficiently low, so that two orthologous regions will be co-assembled in one sequence.

This final reference transcriptome contains 36,459 sequences grouped in 21,888 components. We quantified each sequence using Bowtie2 and eXpress (see Materials and Methods) and assigned a measure of expression to each one. The distribution of the expression levels from all the samples is shown in Fig. S2. There are two modes in this distribution, suggesting that half of the Trinity components are highly expressed, whereas the other half are lowly expressed and could be interpreted as transcription noise, which has been previously reported with transcriptome data^36^.

We further annotated the 21,888 components by aligning them against the *D. mojavensis* genome (see Materials and Methods). We obtained 11,155 components that were unambiguously assigned to a single protein coding gene; 2,109 components matched several protein coding genes; 7,610 components corresponded to intergenic regions; 219 corresponded to TEs; and 795 did not align to the reference genome. The 11,155 components that mapped to unique genes were then clustered into 5,450 genes, for which we have a gene annotation. The 219 components that corresponded to transposable elements were clustered into 69 TE families. The analysis was then performed for 69 TE families and a total of 15,964 genes that corresponded to 5,450 predicted/annotated genes, 2,109 sequences matching several protein coding genes, 7,610 intergenic RNAs, and 795 other sequences not mapping on the reference genome of *D. mojavensis*.

### Expression divergence of the parental transcriptomes

**Figure 2:**
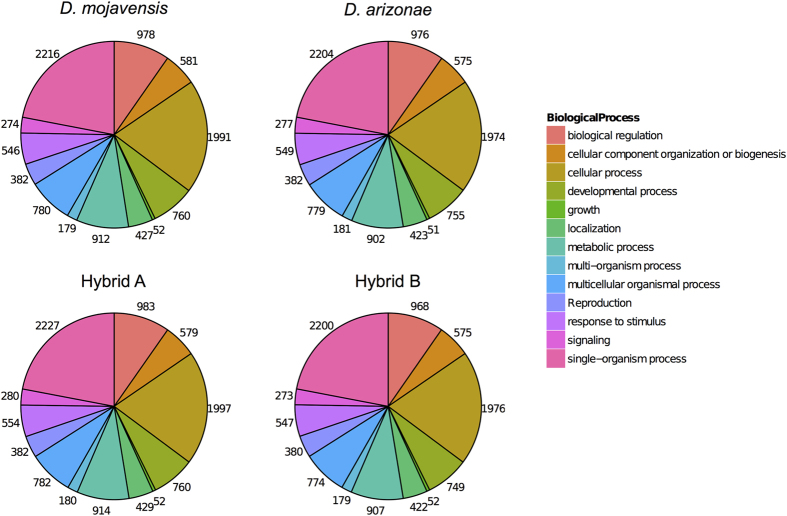
Distribution of the GOterm: Biological Process (level 2). The genes predicted in *D. mojavensis* were assigned the GOterm of the orthologous genes in *D. melanogaster*.

**Figure 3:**
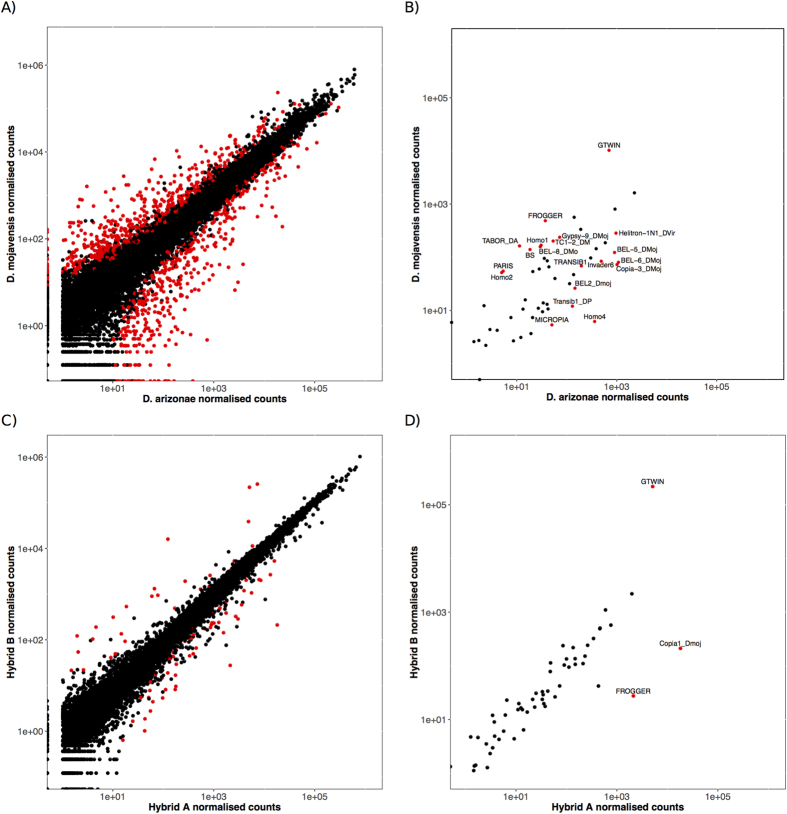
Scatterplots of the normalised reads counts. Scatterplots of the normalised reads counts measured in genes (**A**) and TEs, between *D. mojavensis* and *D. arizonae* (**B**). Scatterplots of the normalised reads counts measured in genes (**C**) and TEs, between hybrid A and hybrid B (**D**). Each dot represents a gene or a TE. Red dots correspond to differentially expressed genes or TEs.

The identified genes for each species and hybrids were classified according to the GO terms. As seen in Fig. 2, the distribution of the GO terms was homogeneous between species and hybrids, which indicates that the same genes were found in the four transcriptomes. Most of the transcribed genes correspond to biological regulation, cellular component and cellular process GO terms. The analysis of the total transcriptome shows that 8% (1,229) of the assembled genes were differentially expressed between *D. mojavensis* and *D. arizonae,* with a maximum fold-change of 209. Of the 1,229 differentially expressed genes between *D. mojavensis* and *D. arizonae*, 624 (51%) corresponded to protein coding regions. Table S2 shows the top 30 differentially expressed genes. Most of these genes have unknown functions based on their orthologs from *D. melanogaster* (23/30). As seen in Fig. 3A and Table S3, the distribution of the fold changes is symmetric, which indicates that a similar number of genes are under- or over-expressed (55% vs 45%) in each species.

From the 69 TE families identified in our data, 20 were differentially expressed between the two parental lines (29%) that belong to the different classes of TEs: eight DNA-transposons (Class II), 11 LTR retrotransposons (Class I), and one non-LTR retrotransposon (Class I) (Fig. 3B, Table S4). As for genes, no asymmetry was detected in the distribution of the fold changes for TEs.

### Transcriptome of the hybrids

Hybrids were obtained in a reciprocal manner, which allowed us to search for parental effects. We found that 89 genes (0.6% of all identified genes from the co-assembling procedure) were differentially expressed between the two hybrid lines (Table S5 and Fig. 3C) with a maximum fold-change of 94 (Table S6). Of these 89 genes, 48 (53%) were annotated as genes and 42% were included in those that were differentially expressed between the parental lines.

From the observation of the fold changes distribution between the hybrids, we suspect a weak asymmetry which could suggest parental effects (Fig. 3C and Table S6). Indeed, 62 genes are over-expressed in hybrid A, whereas 27 are over-expressed in hybrid B (respectively, 69% and 31%). This asymmetry remains true if we restrict the results to genes identified as protein coding genes: 37 (77%) are up-regulated in hybrid A, whereas only 11 (23%) are up-regulated in hybrid B. We tested if this asymmetry in the gene expression of hybrids could be explained by specific parental effects using a linear model. We compared a model in which *Hyb* ~ *a*_*1*_ (*D. mojavensis* + *D. arizonae*)/2 + *a*_*2*_ *(D. mojavensis−D. arizonae*)/2 against a model in which *a*_*2*_ = *0*. We show that the average expression of the parental lines explains most of the variance (R^2^ = 0.9802), meaning that each parental line contributes equally to the hybrid gene expression.

For the TE families, only 3 (4%) are differentially expressed between the two hybrids (Fig. 3D, Table S7), and they were already detected as differentially expressed between the parental lines.

### Expression Inheritance

**Figure 4:**
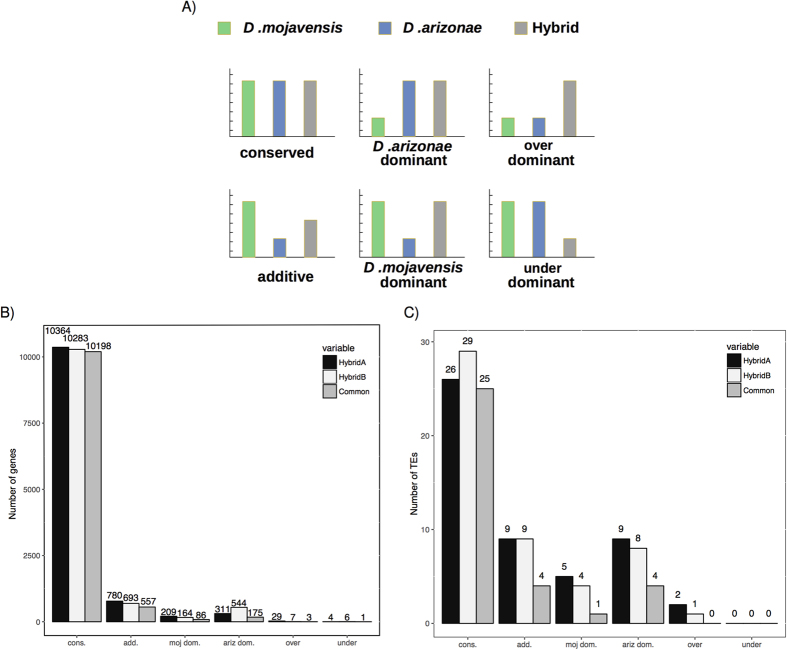
Expression inheritance of genes and TEs. (**A**) Illustration of six patterns of expression inheritance. Genes are considered to have a conserved expression when the expression is not different between the two parental lines and the expression in the hybrid is not different compared to each parental line. Genes and TEs are classified as additive when the expression is different between the two parental lines and the expression in the hybrid is intermediate. Genes and TEs for which the expression is similar to only one parental line, *D. mojavensis* or *D. arizonae*, are classified as *D. mojavensis*-dominant or *D. arizonae*-dominant. Genes and TEs are classified as over-dominant when the expression in the hybrid line is significantly higher than in both parental lines and as under-dominant if the expression is significantly lower than in both parental lines (adapted from MacManus *et al*. 2010). For each gene or TE, two conditions are significantly different when the FDR is below 0.01 and the fold-change is higher than 1.5 (**B**) Expression inheritance of genes. (**C**) Expression inheritance of TEs.

We determined the mode of expression inheritance for the genes and the TEs by comparing the expression levels between one hybrid and each of the parental lines. The expression inheritance was analyzed according to McManus *et al*.^37^ (Fig. 4A). The genes and TEs are classified in the six different categories of expression inheritance depending on the significance of the differential expression measured by comparing all our conditions pairwise (see Material and Methods). When comparing the expression of one gene, two conditions are significantly different when the FDR is below 0.01 and the fold-change is higher than 1.5; if one of this criteria is not fulfilled, the gene expression will be considered as not different between the two conditions that are compared.

### Gene expression in hybrids is highly conserved

For all genes, the “conserved” category (in which hybrids have the same levels of expression as the parental lines and there is no difference between parental lines) is the most common for both hybrid lines, including 10,364 genes in hybrid A and 10,283 in hybrid B (>88%). The conserved genes in hybrid A and hybrid B are mostly the same (98%) (Fig. 4B, Fig. S3), which indicates that the genes that are not differentially expressed between the parental lines have the same expression in the hybrid lines. Six percent of the genes (780 genes in hybrid A and 693 in hybrid B) follow the additive model, which means hybrid expression is intermediate between both parental lines. Four percent of the genes in hybrid A and 6% in hybrid B follow a dominant model.

We found no massive misexpression of the genes in hybrids. Few genes were classified as over-dominant (29 in hybrid A, 7 in hybrid B) or under-dominant (4 in hybrid A, 6 in hybrid B), of which 20 (43%) were identified as protein coding genes (Fig. 4B, Fig. S3). Only 4 misexpressed genes were common between both hybrids, one under-dominant gene (Table 1), and 3 over-dominant genes (Table 2). Under-dominant genes seem to be related to reproduction while over-dominant genes are related to olfaction and behavior.

### TEs are under control in hybrids

From the 31 expressed TEs that are not differentially expressed between the parental lines, 25 were also not differentially expressed in both hybrids and belonged to the conserved category (Fig. 4C, Fig. S3). Nine elements in the hybrids followed the additive model; 14 elements in hybrid A and 12 in hybrid B were either *D. mojavensis*-dominant or *D. arizonae*-dominant. No element was in the under-dominant category. Two TEs in hybrid A (FROGGER and *Copia1*-Dmoj) and only one in hybrid B (GTWIN) belonged to the over-dominant category. For two of them, Copia1 in hybrid A and GTWIN in hybrid B, the over-expression was especially high, with fold-changes higher than 10 compared to the parental line with the highest expression (see bellow). We have performed RTqPCR for these specific TEs and other genes to validate RNAseq differences in expression (Fig. S4).

### Global TE expression and piRNA amounts

piRNAs are a class of small, non-coding RNAs (23 to 29 nucleotides long) that play a role in the silencing of TEs. piRNAs can be produced in two different pathways: primary piRNAs come from piRNA clusters distributed throughout the genome and are produced in somatic and germline cells, whereas secondary piRNAs are derived from the product of a cleavage of functional TE transcripts and are maternally transmitted to embryos. Secondary piRNA production, also called the “ping-pong” pathway, is characterized by piRNA sequences that present complementarity with exactly 10 nucleotides of the primary piRNA.

In order to understand the regulation of TEs in hybrids, we compared the TE mRNA expression in the hybrids with the piRNA normalized counts for each hybrid we sequenced (Fig. S5A,B). No correlation was observed for either hybrid (IC (r) = [−0.40; 0.23] with 95% confidence in Hybrid A, and IC (r) = [−0.36; 0.26] with 95% confidence in Hybrid B). We also considered the ratio of expression between hybrids, and the ratio of expression between the piRNA normalized counts. We first analyzed all piRNAs together, and again no significant correlation was observed (IC (r) = [−0.33; 0.15] with 95% confidence, Fig. S5C). However, if we consider only secondary piRNAs, we clearly see a significant negative correlation (IC (r) = [−0.58; −0.16] with 95% confidence, the p-value associated to the t-test is 0.0011, Fig. S5C). A closer look at the data reveals that this correlation is essentially driven by GTWIN, Frogger and Copia1_Dmoj and disappears when these 3 elements are removed from the analysis.

Since the production of piRNA is dependent on the efficiency of genes from the piRNA biogenesis pathway, we investigated if the expression of this set of genes was modified in the hybrids. In Table S8, we show that, of the thirty genes that were analyzed, none was differentially expressed between hybrids, and only two were differentially expressed between the parental lines.

### GTWIN, Copia1 and Frogger elements

We determined the copy number and structure of these three TE families in the *D. mojavensis* sequenced genome. GTWIN (which belongs to the *gypsy*-like family) is highly expressed in hybrid B and is present in eight copies in the *D. mojavensis* genome. The average identity between copies (pairwise) was 99%, which indicates that GTWIN insertions are recent in the sequenced genome and may correspond to still active copies. For this element, no SNPs were found along the sequence in the reads of hybrid B or hybrid A, which indicates that only one type of insertion is being transcribed.

The *Copia1* element, which was significantly more highly expressed in Hybrid A, is present in approximately 40 copies in the *D. mojavensis* genome, with an average identity of up to 70%, which indicates that the elements were probably active at a more distant time and that the transcripts are from the most intact copies. For *Copia1* element, only two SNPs were identified along the sequence in hybrid A, which indicates that at most two types of insertions are being transcribed.

The *Frogger* element, which was also significantly more expressed in Hybrid A, is present with 10 copies in the *D. mojavensis* genome, with an average identity between copies of up to 79%. Moreover, we were able to reconstruct 24 potential sequences from the transcriptome, indicating that more than one variant is being transcribed.

**Figure 5:**
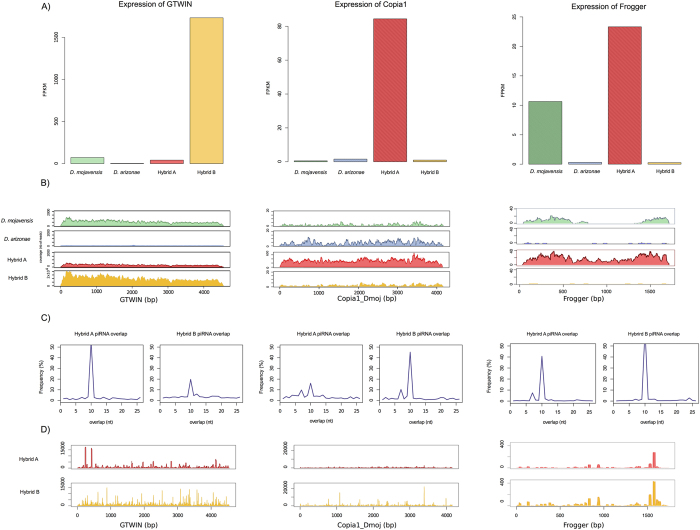
Description of *GTWIN* (left), *Copia1* (middle) and Frogger (rigth). The expression (**A**) and coverage (**B**) of the TEs for each parental line and hybrid. (**C**) The overlapping frequency of piRNA for both hybrids. A peak in the frequency for an overlapping size of 10 nucleotides is characteristic of a ping-pong amplification cycle. The height of the peak indicates the proportion of piRNAs implicated in the ping-pong cycle. (**D**) piRNA coverage of the TEs for both hybrid lines.

To better understand the expression increase of these TEs in hybrids, we specifically analyzed the piRNAs from Hybrid A and B and searched for ping-pong signatures for GTWIN, *Copia1* and *Frogger* (Fig. 5)^20^,^38^.

For the three elements, the high levels of mRNA is accompanied by a weak ping pong signature in the piRNA pool (Fig. 5A–D and Fig. S5), which is compatible with the hypothesis that no secondary piRNAs were maternally transmitted to silence the element in the germline.

## Discussion

Eight percent of the genes were differentially expressed between the two parental lines, *D. mojavensis* and *D. arizonae*, which diverged between 0.6 and 1 Mya ago^28^,^31^,^32^,^39^. Studies comparing more distant species, such as *D. melanogaster* and *D. sechellia*, which diverged approximately 1.2 Mya ago^40^, showed that up to 78% of genes were differentially expressed^36^. In other studies comparing *D. melanogaster, D. simulans* and *D. yakuba*^41^,^42^, at least 27% of the genes were differentially expressed between species or strains. Genes that were differentially expressed between the parental lines were essentially related to development and reproduction. The divergence in gene expression that we observed may reflect the low divergence time between the two species. However, an alternative hypothesis could be related to differences across tissue samples. Indeed, Gomes and Civetta (2015) worked with species with a similar divergence than ours, and have found that 15% of genes were differentially expressed in male reproductive tract tissues^43^. This could be related to the faster male hypothesis that predicts differences across tissue samples in rates of evolution^44^. Future work on the male germline expression could help to test this hypothesis.

We performed reciprocal crosses to check for parental effects on hybrids between *D. mojavensis* and *D. arizonae*. In general, gene expression was fairly similar between the hybrids, with fewer genes differentially expressed than between the parental lines. Moreover, for the 0.6% of genes that differed between the hybrids, most were up-regulated in hybrid A. This indicates that for a few genes there is an effect of the *D. arizonae* parental line. In the study by Gomes and Civetta (2005), in which male reproductive tract expression from reciprocal crosses was analyzed, they found significantly more misexpressed genes in the sterile hybrid than the fertile one^43^. In our case, females are not sterile in either of the crosses, which could explain the low number of differentially expressed genes. Most of the other previous studies on hybrid crosses were performed in one cross direction^45^.

In hybrids between *D. melanogaster*/*D. sechellia* and *D. melanogaster*/*D. simulans*, most of the genes were either *sechellia/simulans*-dominant or under-expressed^37^,^41^. In our study, the comparison between the hybrids and the parental lines showed that most of the genes had an expression that was conserved or additive, as previously obtained for fertile hybrids obtained with *D. pseudoobscura* species^43^. In our case, we analyzed female hybrids that are not sterile. One other hypothesis that could explain the low number of misregulated genes could be low divergence between the parental species, but this needs to be tested by the analysis of other tissue, such as testis, since the rates of evolution can be tissue specific. Few genes were up- or down-regulated. The detailed analysis of these unregulated categories shows that the genes are related to reproduction, development and behavior. In a previous study, different life history traits and viability were measured in hybrids of *D. mojavensis* and *D. arizonae* and were compared to their parents^32^. Female hybrids (from both crosses) had performances equal to the one of their mothers. This is consistent with our observation because the vast majority of the genes had a conserved pattern between hybrids and parents. Moreover, genes that are up-regulated in hybrids may be involved in the good performance of the hybrids. In contrast, down-regulated genes are related to reproduction and could preclude sterility problems in the hybrids.

The comparison of the expression between *D. mojavensis* and *D. arizonae* showed that of the 69 TEs that were identified in the transcriptome, 29% were differentially expressed. This emphasizes the fact that closely related species may have very different amounts and expression levels of TEs^46^,^47^,^48^,^49^ and that these differences may also exist between strains^5^,^49^. Again, when comparing both hybrids, very few elements were differentially expressed, indicating that regulatory systems are operating in the hybrids. This has not been observed in hybrids between more distantly related species. In crosses between *D. melanogaster* and *D. simulans*, which were performed with specific mutant strains of *D. simulans* that “allow” the development of the F1 hybrids, a massive increase of transcription was observed for most of the TEs. Kelleher *et al*. (2012) claimed that time allowed for divergence in the regulation system, namely the divergence of the proteins of the piRNA biogenesis, that were no longer efficient in the silencing of TEs^50^. In another Drosophila model, with hybrids between *D. buzzatti* and *D. koepferae*, Vela *et al*. (2014) showed, in a genome-wide manner, massive rearrangements in the F1 hybrids^24^. In both systems, a wide variety of TEs were responsible for most of the genomic instability in the hybrids. For the *D. mojavensis* and *D. arizonae* species used in this study, the divergence between orthologous genes is less than 2% for more than 95% of the genes, and we did not observe any differential expression in hybrids for genes from the piRNA biogenesis pathway, which could explain some weakness on the silencing of TEs. In our analysis, we identified three TEs (SM Table 7) belonging to the LTR class that were differentially expressed between hybrids, and up-regulated compared to the parents. GTWIN is highly expressed in hybrid B, *Copia1* and *Frogger* are highly expressed in hybrid A. The specific analysis of RNA sequences from these elements allows us to propose a scenario that is consistent with the idea of clusters producing piRNAs that are not equally present in the parental lines. A GTWIN insertion could be present in the paternal line of hybrid B in *D. mojavensis*, but not in the maternal line because the expression of GWTIN is low in *D. arizonae*; therefore, the secondary piRNA corresponding to the element could not be transmitted by the maternal line and did not lead to a ping-pong amplification cycle in hybrid B. The same scenario can be proposed for *Copia1*. The *Copia1* insertions could be present in the paternal line of hybrid A in *D. arizonae*, but not in the maternal line because the expression of *Copia1* is low in *D. mojavensis*. Therefore, the secondary piRNA corresponding to the element could not be transmitted by the maternal line and did not lead to a ping-pong amplification cycle in hybrid A. This scenario corresponds to what is observed when crossing different strains of *D. melanogaster, D. simulans* and *D. virilis* harboring different TE amounts and activities, which results in the derepression of TEs^10^,^16^,^21^,^51^,^52^. The Frogger situation seems to be more complex and it’s probably related to the diversity of copies that are expressed in the parental genomes. We could expect to have a preferential silencing in the hybrid A, and this was not observed. We are probably being limited by the capacity to attribute transcripts and piRNA to specific insertions.

In crosses between *D. melanogaster* that induce hybrid dysgenesis, advances have been made that show that the absence of maternally transmitted piRNAs from specific TEs is responsible for hybrid female gonadic atrophy or sterility. Instead, our results suggest that the female germline is successfully protected (even if some specific elements escape this control) against transposition by the maternally transmitted secondary piRNAs. However, TE expression variation between reciprocal hybrids in the female germ line stresses the necessity of further population studies in order to investigate whether these mobile elements might contribute to post-zygotic reproductive isolation between *D. mojavensis* and *D. arizonae*. We have shown that some specific TEs are upregulated in the male germline depending on the source population of males and females, and the direction of the cross^53^. Thus, sterility in males could be associated with the mobilization of TEs and a transcritome analysis of the male germ line is needed because it could explain why TEs, despite a strong negative selection against deleterious transposition effects, remain successfully active in the male line. Population studies on TEs in this species pair can give insights into how reproductive isolation evolves.

We showed that the *D. mojavensis* and *D. arizonae* parental lines differ in their gene expression (~8% of the genes are differentially expressed) and in their TE expression (~29% of the TEs are differentially expressed). Reciprocal hybrids presented average levels of gene expression compared to the parental lines, with some specific gene categories being misexpressed, such as genes related to reproduction and development. As for TEs, we identified few families that were strongly expressed in hybrid crosses, relative to the parental lines. Moreover, the piRNA sequencing confirmed that in hybrids, the deregulation of specific TE families is due to the absence of such small RNAs. We therefore propose that TEs can indeed be major players on genome differentiation and be involved in the first steps of genomic incompatibilities through small RNA regulation.

## Methods

### Drosophila strains and RNA sequencing

We sequenced polyA+ RNA from the ovaries of flies. The sequenced strains were *D. mojavensis,* from the Anza Borrego Desert, CA (stock number: 15081-1352.01) and *D. arizonae*, from Metztitlan-Hidalgo, Mexico (stock number: 15081-1271.17), both obtained from the US San Diego Drosophila Stock Center. These are two allopatric species with which we can perform reciprocal crosses in laboratory conditions to provide sufficient F1 hybrid individuals to obtain enough RNA for sequencing. Parental individuals were separated to collect virgins one day after hatching. Crosses were performed with 3-day-old flies; ten males and eight females were placed in 2.3 × 9.5 cm tubes containing culture medium under the same temperature and humidity conditions. Virgin female parental flies and F1 female hybrids were collected after hatching, at one day of age, and were isolated until they reached ten days. RNA was extracted from the ovaries of 10-day-old flies (i.e., *D. mojavensis, D. arizonae* and hybrids from reciprocal crosses). The extractions were performed using the RNeasy kit (Qiagen), and the samples were then treated with DNase (DNA-free Kit, Ambion) and stored at −80 °C. The samples were quantified by fluorescence in a Bioanalyzer 2100 (Agilent). The crosses were generated in three independent biological replicates. For each replicate, and when possible, the extracted RNA was divided into two parts to generate two cDNA libraries (two replicates per condition). If no difference was observed between the technical replicates, the reads were pooled. RNA was sequenced by Illumina Technology in Illumina HiSeq 2000 and Illumina HiSeq3000 by the GenoToul facility (Toulouse, France). One sample was sequenced in 2 × 51 bp reads and the medium size of the inserts was 300 bp. The other two were sequenced in 2 × 125 bp reads, with the same size of the insert. We used UrQt^54^ with the default parameters to remove the low quality bases and the polyA tails.

### Assembly of the transcriptome

The reads were co-assembled, *i.e.,* we used the reads from all (parental and hybrid) lines that passed the quality filter to construct a *de novo* reference transcriptome. We ran Trinity^55^ version r2013_08_14 with the default parameters and a group_pairs_distance of 600. Thus, these transcripts are consensus transcripts. The procedure is described in Fig. S1). From the Trinity assembler we obtained several sequences. Some of them correspond to the same gene, for example due to alternative splicing events, and Trinity will consider them as a component (for example component1 will have sequence 1 and 2). Moreover, Trinity may also produce an independent sequence from the same gene (that will be named component2, with sequence 1). The alignment of the sequences against a reference genome allow to merge components that belong to the same gene.

This approach is possible because the two parental lines diverged recently, so we assumed that the transcripts of the species and the hybrids are similar enough to be assembled together. This method has the effect of increasing the sequencing depth and allows us to better assemble transcripts that are too lowly expressed in one or more species and that could not be assembled otherwise, which can be the case for TEs, which can be lowly expressed in parental lines. Additionally, unlike the mapping method, this approach has no bias in favor of *D. mojavensis*.

To validate this approach, we compared the results obtained by the co-assembly with those obtained from the single assemblies of each species and hybrids. We aligned the components obtained from the single assemblies to the one from the co-assembly and considered them associated if they mapped with at least 80% identity with 80% of query coverage (the query corresponds to the components from the single assemblies). For the single assemblies and the co-assembly, we estimated the number of chimeric components by the number of components that did not align on the reference genome of *D. mojavensis* with at least 80% identity and 80% of query coverage.

### Quantification of expression

The quantification of the gene expression of each replicate of each line was performed with Bowtie2^56^ and eXpress^57^. Bowtie2 (with –local –all -N 1) was used to map the reads to the genes and TEs of the reference transcriptome we assembled. The number of reads aligning against each sequence was then counted by eXpress, which provided access to the expression of the transcripts and the genes (in FPKM). eXpress also addresses multiple mapping and assigns the read to its most likely location. We performed RTqPCR experiments on a selected set of TEs and genes that are differentially expressed or not (Fig. S3).

### Gene and TE identification

To identify the genes among the components assembled by Trinity, we downloaded the 15,179 sequences of annotated and predicted genes from *D. mojavensis* (version r1.3 from http://flybase.org/) and aligned our components with BLAT^58^ with at least 80% identity and with a minimum query coverage of 80%. We also aligned all of the components with BLAT to the reference genome of *D. mojavensis* (version r1.3 from http://flybase.org/) with at least 80% identity and with a minimum query coverage of 80% to search for transcripts originating from the intergenic region.

To the genes predicted in *D. mojavensis,* we assigned the GO term of the orthologous genes in *D. melanogaster* using the orthologous tables downloaded from http://flybase.org/. We also ran Blast2GO^59^ on the assembled transcripts and obtained the GO term for the transcripts. We kept all of the GO terms provided by at least one of the methods.

For the TE identification, we used BLAT to align our sequences against consensus TEs from Repbase Drosophila^60^ (2,296TEs) and against a homemade database (4575 TEs). The homemade database was generated by running Repeatmasker^61^ (http://www.repeatmasker.org/) on the *D. mojavensis* reference genome. We kept the alignments with an identity percentage higher than 70%, and with a minimum query coverage of 80%. Eighteen of the 69 TEs are lowly expressed in all species and hybrids (*i.e.* the normalized counts are <5 for each species and hybrids), as are another 4,267 genes of the total of 15,964. These genes were included in the analyses but were not tested for differential expression and therefore were not considered in the analyses of expression inheritance. Eight other genes were identified as mitochondrial genes (4–5 million reads per replicate) and were not included in our analyses.

The assembler may produce several transcripts that correspond to the same gene or to the same transposable element (Fig. S1). For instance, when a gene has a low expression level, some of the genes can be lowly covered or not covered at all by the reads, and the assembler will fail in the reconstruction of the complete gene but may assemble some parts of it (Fig. S1). This step also allows us to cluster some components together as they map to the same gene or transposable element.

### Differential Expression with DESeq

We used DESeq2^62^, an R package, to identify genes and TEs that were differentially expressed between two lines. DESeq2 normalizes counts using so-called size factors that are estimated according to the median counts taken on all genes. The underlying assumption is that most genes do not vary from one condition to another, which implies that the median expression value is constant (thus giving a proper ground to obtain comparable values). In our case, we expect this condition to be fulfilled. After the step of normalization, DESeq2 estimates the means and variances of raw read counts and tests for differential expression based on a model using the negative binomial distribution. Genes and TEs are classified as significantly differentially expressed if: (1) the p-value, after correction for multiple tests with the False Discovery Rate (FDR), is below 0.01; and (2) the fold-change (expression ratio between the compared conditions) is above 1.5. Genes and TEs were considered to be too lowly expressed in all conditions when the normalized counts for each line did not exceed 5. These genes and TEs were excluded from the analyses.

### RT-qPCR proof of expression

The levels of expression of *Copia-1*, GTWIN, Frogger, I, Invader, and for the gene #484367, #20074 and #28887 were validated by RTq-PCR. Primers were designed from the consensus obtained after the transcriptome assembly and were specific to our strains. One microgram of sequenced RNA was treated with DNase (DNA-free Kit, Ambion) and was converted to cDNA using a Thermoscript Invitrogen kit. The cDNA was diluted 50 times, and the relative mRNA level was quantified using SYBR green qPCR in a LightCycler 480 instrument (Roche Diagnostics). The RT–qPCR experiments were performed with two to three biological replicates. Only RT–qPCR experiments with efficiencies greater than 1.9 were retained. The following primers were used: GTWIN forward 5′-CGC TGA CGG CAA TAA TGA AAG C-3′ and GTWIN reverse 5′-ATC TTC CGA TGC CAA GAT A-3′; Copia1 forward 5′-GTG GAC CTA TAA GGC AAG TAT C-3′ and Copia1 reverse 5′-AGA CCT TTC TGA CGC TCT A-3′; Frogger forward 5′-GTCTCGGATGTCATTTGCCC-3′ and Frogger reverse 5′-ACCCGATATTTTGCACGCAG-3′; I forward 5′-TCGACATTACGGCAACAGAAC-3′ and I reverse 5′-TCGTGATGAGTCCGGTTTCT-3′; Invader forward 5′-CCTTGCCTCTGTTTGCTGTT-3′ and Invader reverse 5′-AGTGGCCATAAGATCGCTGA-3′; 484367 forward 5′-ATCGGTCGAGAAAGTCCTCC-3′ and 484367 reverse 5′-AGTCTGGTTGGGTATGTGCA-3′; 20074 forward 5′ –ATCGTGCTCCTATGATCGCA-3′ and 20074 reverse 5′-GGCATCCCAACTACCGTACT-3′; 22887 forward 5′-CGACGCACAATACCAACGAT-3′ and 22887 reverse 5′-TGCCGTCGAATTATTGCCTG-3′.

The relative expression levels of the elements or genes were measured with the constitutive expression of the endogenous ribosomal gene 49 (rp49), also known as asnrpL32^63^,^64^.

### Small RNA extraction and sequencing

Small RNAs from the hybrid A and hybrid B ovaries were manually isolated on HiTrap Q HP anion exchange columns (GE Healthcare) as described in ref. 65. The library construction and 50 nt read sequencing were performed by Fasteris SA (Switzerland) on an Illumina HiSeq 2500 instrument.

### Analyses of piRNA, ping-pong signatures and identification of ping-pong partners

We considered as piRNA the sequences of small RNAs of length 23 to 29 nt that could be aligned against TEs from our assembled transcriptome or against TEs found in the genome of *D.mojavensis* (see TE annotation above). The alignments were performed with Bowtie using the –very-sensitive option. We then used the “Mississippi Tools”^66^, which search for ping-pong signatures by counting the number of pairs of piRNAs overlapping for 1 to 26 nucleotides.

## Additional Information

**How to cite this article:** Lopez-Maestre, H. *et al*. Identification of misexpressed genetic elements in hybrids between Drosophila-related species. *Sci. Rep.* **7**, 40618; doi: 10.1038/srep40618 (2017).

**Publisher's note:** Springer Nature remains neutral with regard to jurisdictional claims in published maps and institutional affiliations.

## Supplementary Material

Supplementary Information

## Figures and Tables

**Table 1:** List of genes under-expressed in hybrid A or hybrid B.

Gene ID or component name	D. melanogaster orthologous	Function*	under express in
FBgn0141780	FBgn0034435	multicellular organism reproduction; neurogenesis	Hybrid A and Hybrid B
FBgn0137150	FBgn0002576	eye morphogenesis	Hybrid A
FBgn0141856	FBgn0028743	regulation of G-protein coupled receptor protein signaling pathway	Hybrid A
comp24320_c1	—	—	Hybrid A
FBgn0142875	FBgn0033307	—	Hybrid B
FBgn0143905	FBgn0053680	—	Hybrid B
comp21992_c0	—	—	Hybrid B
comp20689_c0	FBgn0038395	multicellular organism reproduction	Hybrid B
comp22566_c0	FBgn0000158	oogenesis; germ-line stem cell population maintenance germarium-derived female germ-line cyst formation	Hybrid B

^*^From flybase.org.

**Table 2:** List of genes over-expressed in hybrid A or hybrid B.

Gene ID or component name	D. melanogaster orthologous	Function*	over express in
FBgn0132927	FBgn0053207	long-term memory; learning or memory; olfactory learning; behavioral response to ethanol	Hybrid A and Hybrid B
comp16418_c0	—	—	Hybrid A and Hyrbrid B
comp23925_c6	—	—	Hybrid A and Hyrbrid B
FBgn0133446	FBgn0038247	open tracheal system development;	Hybrid A
FBgn0134214	FBgn0265296	neuron projection morphogenesis; homophilic cell adhesion via plasma membrane adhesion molecules	Hybrid A
FBgn0136788	FBgn0053653	sensory perception of pain; synaptic transmission, glutamatergic dense core granule exocytosis; synaptic vesicle exocytosis;	Hybrid A
FBgn0137587	FBgn0029521	olfactory behavior; sensory perception of smell	Hybrid A
FBgn0138472	FBgn0030989	intercellular transport	Hybrid A
FBgn0143279	FBgn0261269	motor neuron axon guidance; open tracheal system development; regulation of tube length, open tracheal system; regulation of tube size, open tracheal system	Hybrid A
FBgn0143673	FBgn0086604	lateral inhibition	Hybrid A
FBgn0143900	FBgn0054038	—	Hybrid A
FBgn0146454	FBgn0031907	trehalose biosynthetic process	Hybrid A
comp18846_c0	—	domain found–no annotation	Hybrid A
comp21244_c0	—	domain found–no annotation	Hybrid A
comp22234_c9	—	—	Hybrid A
comp23342_c9	—	—	Hybrid A
comp5990_c0	—	domain found–no annotation	Hybrid A
comp15705_c0	—	—	Hybrid A
comp16249_c1	—	—	Hybrid A
comp16289_c1	—	—	Hybrid A
comp16843_c0	—	—	Hybrid A
comp16843_c1	—	—	Hybrid A
comp18846_c2	—	—	Hybrid A
comp21588_c6	—	—	Hybrid A
comp22885_c15	—	—	Hybrid A
comp23090_c13	—	—	Hybrid A
comp23090_c5	—	—	Hybrid A
comp23342_c6	—	—	Hybrid A
comp23819_c0	—	—	Hybrid A
FBgn0136851	FBgn0024913	cell growth; dendrite morphogenesis	Hybrid B
comp16722_c1	—	Expansin/pollen allergen, DPBB domain (IPR007112)	Hybrid B
comp18737_c0	—	—	Hybrid B
comp23750_c1	—	domain found–no annotation	Hybrid B

^*^From flybase.org.
