# Supplementary material for: Identification of misexpressed genetic elements in hybrids between Drosophila-related species
Source: Sci Rep. 2017 Jan 16;7:40618. doi: 10.1038/srep40618 (PMC5238404; doi:10.1038/srep40618)
Supplement: Supplementary Information [file srep40618-s1.pdf]

Title: **Identification of misexpressed genetic elements in hybrids between Drosophila-related species**

Hélène Lopez-Maestre[1,2], Elias A. G. Carnelossi[3], Vincent Lacroix[1,2], Nelly Burlet[1], Bruno Mugat[4], Séverine Chambeyron[4], Claudia M. A. Carareto[3], Cristina Vieira[1]*

Supplementary Material

Supplementary Figures

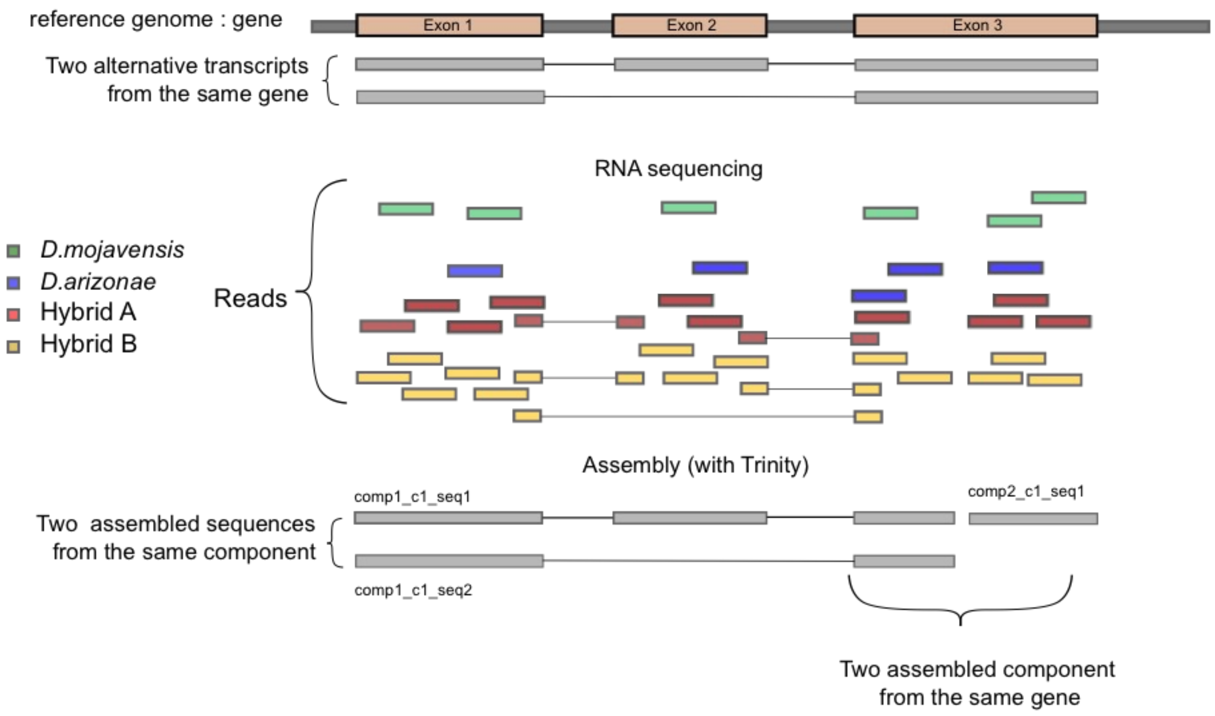

Supplementary Figure 1: Co-assembly result, example on one gene.

In this example, a gene has two alternative transcripts. Due to the coverage heterogeneity in RNAseq data, there is a lack of reads in the third exon of the gene. Thus the assembler fails in the reconstruction of the transcripts and assembled two components for one gene. The first component has two alternative sequences that cover the splicing event present in the real transcripts.

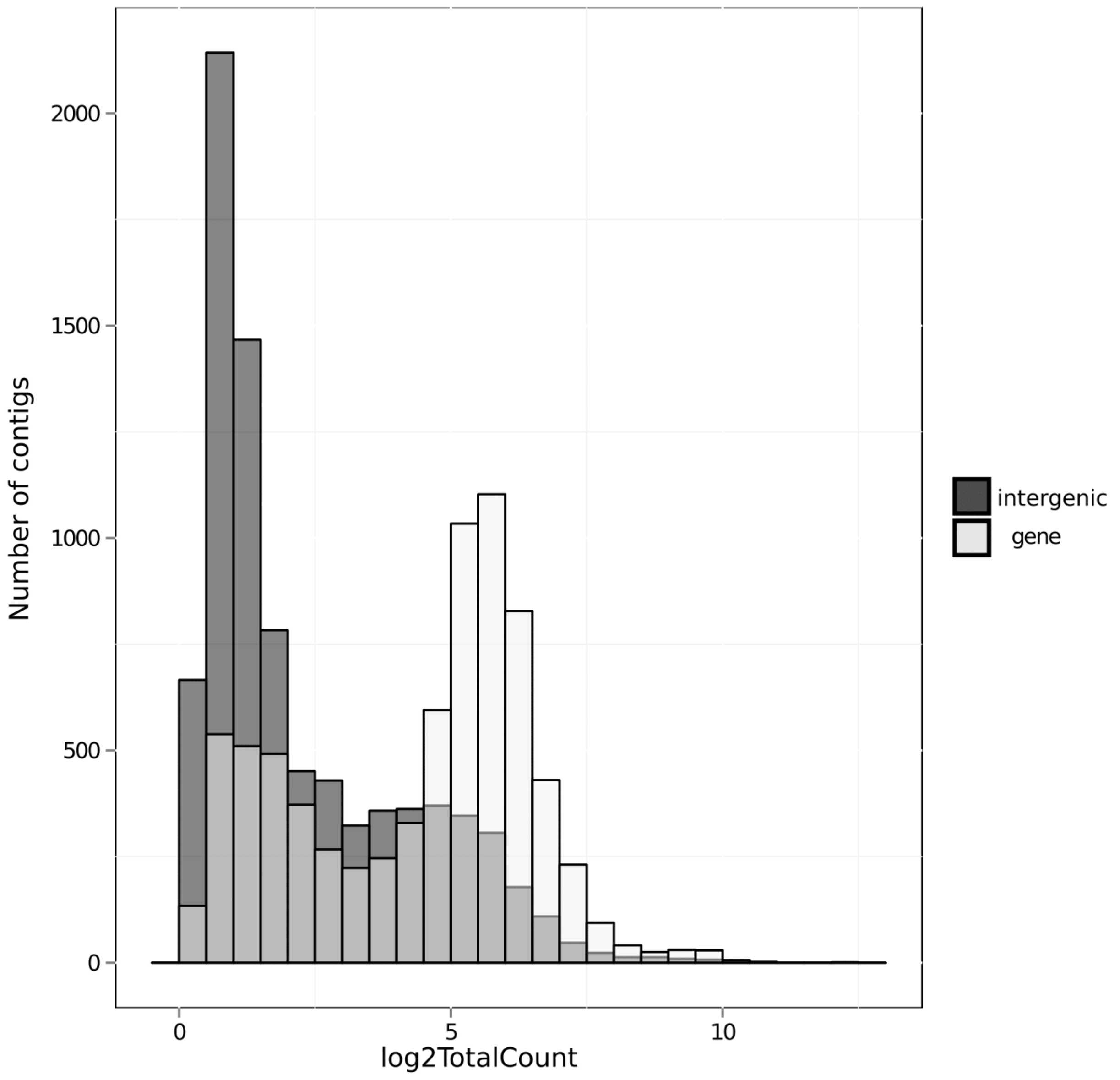

Supplementary Figure 2: Distribution of the total expression from all the samples (in log2FPKM) of the assembled components corresponding to protein-coding genes (white) or components corresponding to potential non coding RNA (darkgrey). There are two modes in this distribution, suggesting that half of the genes are highly expressed, whereas the other half are lowly expressed and could be interpreted as transcription noise, which has been previously reported with transcriptome data.

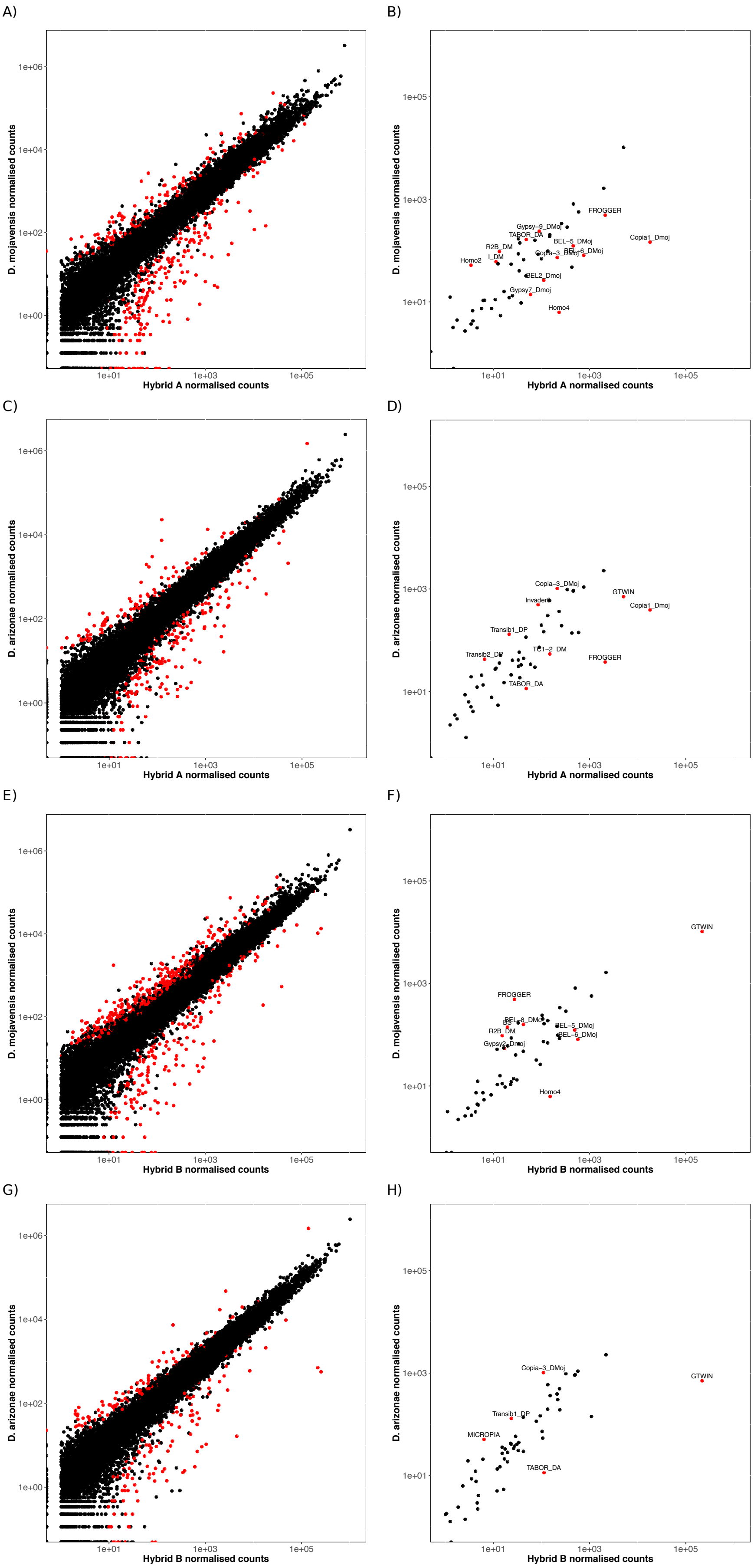

A)
B)
C)
D)
E)
F)
G)
H)
D. mojavensis normalised counts
D. arizonae normalised counts
Hybrid A normalised counts
Hybrid B normalised counts
1e+00
1e+01
1e+02
1e+03
1e+04
1e+05
1e+06
FROGGER
Gypsy-9_DMoj
TABOR_DA
Copia1_Dmoj
BEL-5_DMoj
R2B_DM
Homo2
I_DM
BEL-6_DMoj
BEL2_Dmoj
Gypsy7_Dmoj
Homo4
Copia-3_DMoj
GTWIN
Invader
Transib1_DP
Transib2_DP
TC1-2_DM
Gypsy2_Dmoj
MICROPIA

Supplementary Figure 3: Scatter plot of the mRNA normalized counts of the parental line vs the hybrids, for genes (left A, C, E, G) and transposable elements (rigth B, D, F, H). Each dot represents a gene or a TE. Red dots correspond to differentially expressed genes or TEs.

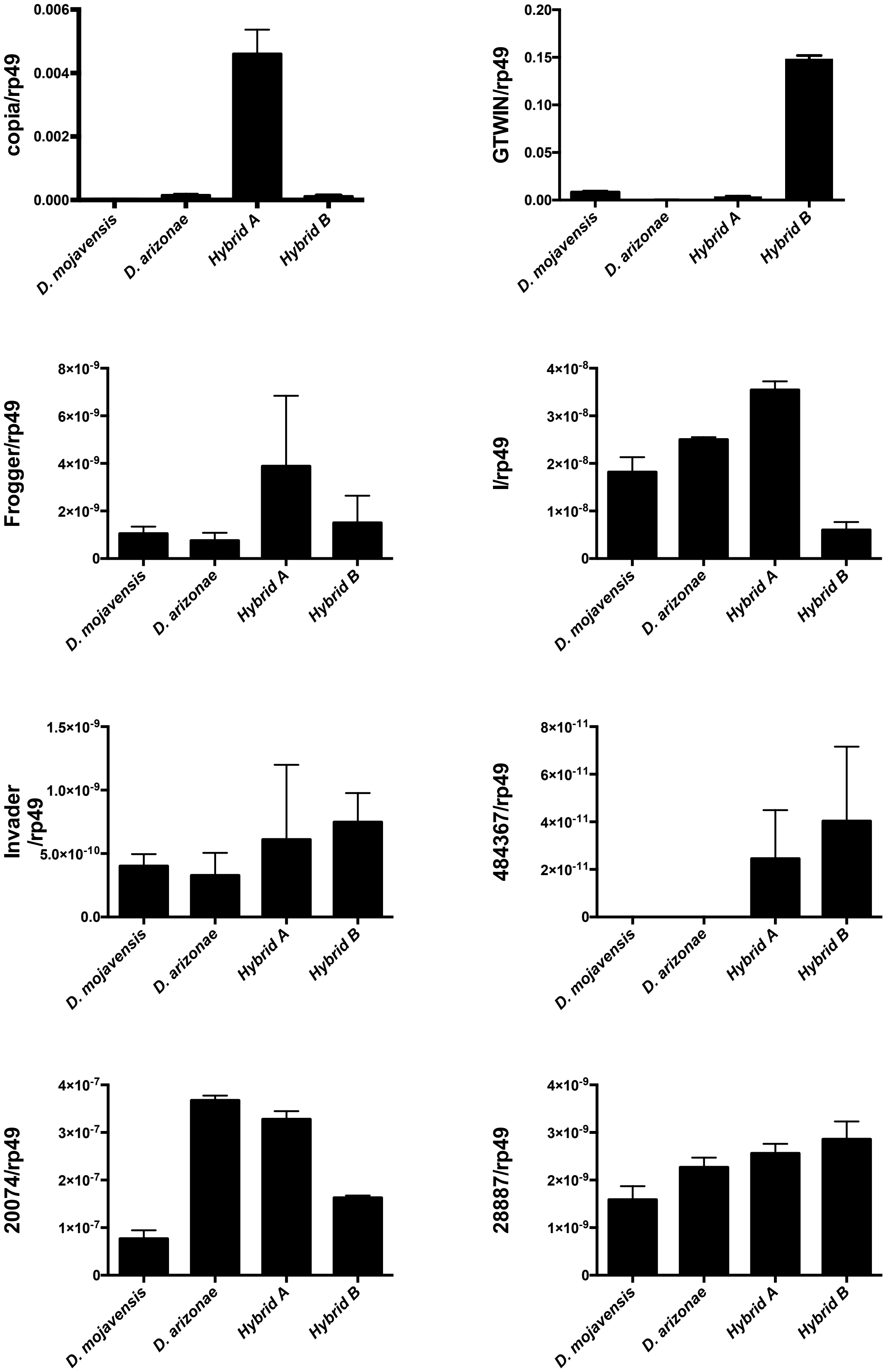

Supplementary Figure 4: RTq-PCR experiments for Copia, GTWIN, Frogger, I and invader TEs, and for genes #48436, #20074 and #2887 in parental lines and hybrids. The results were in agreement with the differential expression obtained with the RNAseq data.

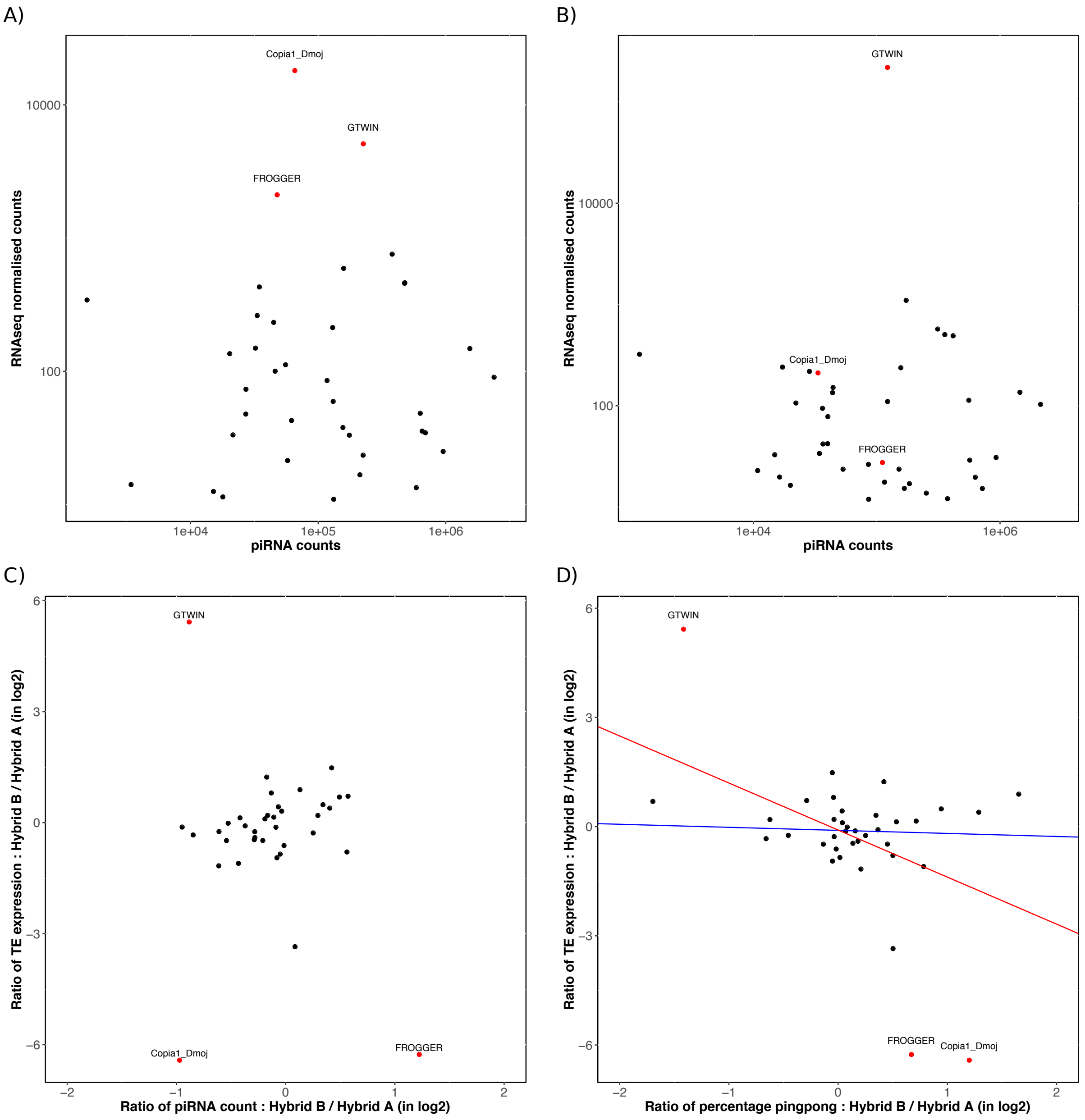

1

Supplementary Figure 5: piRNA analysis. Scatter plots representing le relation between piRNA amount and mRNA from TE (A- hybrid A, B hybrid B). Ratio between hybrid A/B for total piRNA (C). Ratio between hybrid A/B for secondary piRNA (C). Dots represent TEs. Red dots indicate the ones that are differentially expressed between hybrid A and B. The red regression line panel D suggest a negative correlation between the ratio of mRNA and the ration of secondary RNA (IC (r) =[-0.58 ; -0.16] with 95% confidence, the p-value associated to the t-test is 0.0011), that disappears when data from GTWIN, copia and frogger are

removed (blue line).

Supplementary tables

Supplementary Table 1: Sequences obtained by Trinity with a co-assembling procedure or without co-assembling for the four transcriptomes.

	Co-assembly	Split assembly			
		D. mojavensis	*D. arizonae*	HybridA	HybridB
# of components	21889	15807	15521	15352	14556
# of components not aligned on D. mojavensis reference genome[a]	815 (4%)	728 (5%)	1227 (8%)	908 (6%)	872 (6%)
N50[b]	2695	2562	2664	2630	2636
Coverage of the genome[c]	24.0 Mb	19.6 Mb	19.6 Mb	19.3 Mb	18.6 Mb
Mapping Back Rate[d]	98.5	98.5	98.5	98.1	98.3

a) Number of components (and %) that do not align on the reference genome of D. mojavensis with 80% of identity and 80% of their length (QC). This may correspond to chimeric sequences.
b) N50 of the assembly: The N50 length is the shortest sequence length at 50% of the assembled sequences.
c) To calculate the total length we take into account only the longest sequence per component assembled by trinity.

d) The mapping back rate corresponds to the proportion of reads mapping back to the assembled transcriptome. For the co-assembly we mapped all the reads from all the species and hybrids back to the transcriptome. For the single assemblies we mapped back only the reads from the corresponding species or hybrid.

Supplementary Table 2: Top 30 genes differentially expressed between the parental lines.

Gene or component name	Fold Change	FDR	UP	
comp15874_c0	208,6	2,5E-66	Moj	-
comp56409_c0	127,6	1,8E-52	Moj	FBgn0051075 (pyruvate metabolic process) FBgn0051076
comp21101_c1	121,3	9,9E-41	Arz	-
comp20866_c17	112,9	2,5E-32	Arz	-
comp3770_c0	111,6	1,7E-39	Arz	-
comp23924_c0	102,4	8,9E-33	Arz	-
comp23663_c0	98,9	3,8E-61	Moj	-
comp23953_c0	88,7	9,8E-79	Arz	-
FBgn0134916	80,0	5,5E-33	Moj	-
FBgn0141615	75,7	5,4E-41	Moj	FBgn0259247 (laccase 2 , chitin-based cuticle development)
comp15637_c0	70,3	5,6E-60	Moj	FBgn0041241 (sensory perception of taste)
comp20866_c10	66,5	3,3E-27	Arz	-
comp129_c1	66,0	3,3E-27	Moj	-
comp129_c0	61,0	3,6E-23	Moj	-
comp20327_c1	59,0	1,5E-29	Moj	-
comp15675_c1	57,8	1,7E-30	Moj	-
comp15950_c0	56,2	6,2E-39	Moj	FBgn0019982 (wound healing, metabolic process)<br><br>FBgn0040705 (mitochondrial electron transport, NADH to ubiquinone)
comp22800_c7	53,8	7,5E-37	Arz	FBgn0264908 (neurogenesis)
FBgn0141106	53,4	4,5E-25	Arz	FBgn0033058 (neuropeptide signaling pathway)
comp17207_c1	51,1	6,3E-24	Arz	-
comp410692_c0	49,8	1,5E-20	Arz	-
comp3221_c0	49,6	6,7E-22	Arz	-

comp19601_c0	47,2	5,4E-24	Arz	-
FBgn0138205	45,9	1,2E-35	Moj	FBgn0265413
comp22637_c8	45,8	2,2E-18	Moj	-
comp20485_c2	43,4	3,9E-25	Arz	-
comp18701_c0	43,2	4,3E-35	Moj	FBgn0032536 (protelysis)<br><br>FBgn0051716 (regulation of JAK-STAT cascade, centrosome organization)
comp3149_c0	42,7	1,4E-18	Arz	-
comp19494_c0	42,3	5,1E-19	Arz	FBgn0030699 (adult somatic muscle development, regulation of transcription)<br>FBgn0261545 (determination of adult lifespan)<br>FBgn0031897<br><br>FBgn0040005 (regulation of GTPase activity)
comp21818_c14	41,7	4,5E-20	Arz	-

Supplementary Table 3: Genes differentially expressed between the parental lines

	Total	Up in *D.mojavensis*	Up in *D.arizonae*
All Genes	1229	684	546
Unique Genes	486	270	166
Multi Genes	138	82	56
intergenic	534	264	270
not in genome	71	18	53

Supplementary Table 4: TEs differentially expressed between *D. mojavensis* and *D.arizonae*

Type of TE	TE Name	Fold Change	FDR	UP
TIR	Homo4	27.7	4.7E-29	Moj
LTR	FROGGER	12.0	1.6E-26	Arz
LTR	Copia-3_DMoj	11.8	1.0E-20	Moj
LTR	GTWIN	11.6	2.3E-19	Arz
LTR	TABOR_DA	10.4	2.5E-15	Arz
LTR	BEL-6_DMoj	9.3	2.6E-10	Moj
TIR	Transib1_DP	8.0	1.2E-07	Moj
TIR	PARIS	6.5	1.0E-05	Arz
LTR	MICROPIA	6.0	8.1E-05	Moj
LTR	BEL-5_DMoj	5.8	2.7E-07	Moj
LINE	BS	5.7	2.1E-05	Arz
LTR	Invader6	5.1	5.5E-06	Moj
TIR	Homo2	4.9	3.9E-03	Arz
LTR	BEL-8_DMo	4.7	1.4E-09	Arz
LTR	BEL2_Dmoj	4.4	1.6E-04	Moj
TIR	Homo1	4.1	3.5E-03	Arz
LTR	TC1-2_DM	3.5	2.5E-09	Arz
LTR	Gypsy-9_DMoj	3.0	2.3E-04	Arz
Helitron	Helitron-1N1_DVir	2.9	5.6E-03	Moj
TIR	TRANSIB1	2.9	5.4E-04	Moj

Supplementary Table 5: Genes differentially expressed between the hybrid lines

	Total	Up in hybrid A	Up in hybrid B
All Genes	89	62	27
Unique Genes	40	32	8
Multi Genes	8	5	3
intergenic	34	19	15
not in genome	7	5	2

Supplementary Table 6: Top 30 genes differentially expressed between the hybrid lines

Gene or component name	Fold Change	FDR	UP	*D.melanogaster* ortholog (function)
comp23953_c0	94.2	5.9E-82	B	-
comp23750_c1	28.2	6.9E-42	B	-
comp16843_c1	15.6	2.6E-08	A	-
comp20770_c4	14.8	1.2E-07	B	-
comp23819_c0	13.6	2.0E-12	A	-
FBgn0136755	13.3	3.7E-08	B	-
FBgn0143900	13.1	3.2E-11	A	FBgn0054038
comp18846_c0	12.2	2.0E-07	A	-
comp20770_c6	12.1	1.6E-06	B	-
comp22800_c7	11.5	4.4E-14	B	FBgn0264908 (neurogenesis)
comp20770_c2	10.8	8.1E-05	B	-
comp16289_c1	9.9	1.2E-05	A	-
comp24148_c0	8.9	5.3E-05	B	-
comp20770_c7	8.7	6.7E-04	B	-
comp21101_c1	8.6	3.6E-08	B	-
comp21244_c9	8.5	9.6E-06	A	-
comp16249_c1	8.1	9.1E-05	A	-
comp24122_c5	8.0	3.3E-04	B	-
FBgn0143905	7.9	5.9E-10	A	FBgn0053680
FBgn0146209	7.7	1.1E-03	A	-
comp15590_c0	7.7	1.4E-03	A	-
comp15705_c0	7.5	2.6E-03	A	-
comp20019_c0	7.4	2.3E-09	B	-
FBgn0134214	7.3	1.6E-14	A	FBgn0265296 (neuron projection morphogenesis)
comp23342_c9	7.1	3.2E-04	A	-
comp16949_c1	6.8	4.6E-03	B	-
comp14298_c0	6.7	3.7E-03	B	-
comp22234_c9	6.6	6.2E-06	A	-
comp18846_c2	6.5	1.3E-03	A	-
FBgn0132849	6.4	2.7E-09	A	FBgn0039201

Supplementary Table 7: TEs differentially expressed between hybrids

Type of TE	TE Name	Fold Change	FDR	normalized counts in Hybrid A	normalized counts in Hybrid B	UP
LTR	FROGGER	62.1	5.5E-76	2110	27	A
LTR	Copia1_Dmoj	55.3	2.7E-38	18053	211	A
LTR	GTWIN	32.7	1.5E-38	5092	218154	B

Supplementary Table 8: Expression data on thirty genes implicated on piRNA biogenesis. None was differentially expressed between hybrids, and only two were differentially expressed between the parental lines. (* p< 0.05).

	Normalised counts in *D.mojavensis*	Normalised counts in *D.arizonae*	Normalised counts in Hybrid A	Normalised counts in Hybrid B	FoldChange between parental lines	FoldChange between hybrids
archipelago	41957	54588	54636	73544	1.3	1.3
Armitage	35341	38519	51264	51201	1.1	1.0
Aubergine	6991	8613	7047	6518	1.2	1.1
Brother_of_Yb	9394	9603	10509	11508	1.0	1.1
cutoff	39168	34880	28427	28756	1.1	1.0
helicase_at_25E	23051	32452	19748	22519	3.2	1.1
Hen1	19535	24719	37437	36735	1.4	1.1
interruptus_cubitus	777	686	759	446	1.2	1.0
Krimper	3106	2415	4889	4319	1.1	1.6
maelstrom	14300	21927	14430	17035	1.3	1.1
minotaur	363	108	129	181	1.5	1.2
PanoramixA	3362	1759	1852	1987	*3.1	1.4
PanoramixB	3157	4638	4468	4910	1.8	1.1
piwi	39997	62132	48770	50212	1.4	1.1
qin	9917	15763	17512	14859	1.5	1.0
shutdownA	4736	5394	4053	4476	1.6	1.2
shutdownB	2850	3851	2838	2840	1.1	1.1
Sister_of_Yb	3254	1060	2752	1829	1.3	1.0
spindle_E	11133	12011	14216	15057	2.7	1.4
tapas	14454	15546	18392	22548	1.1	1.1
tejas	4499	3708	4100	4505	1.1	1.2
tudor	14	1	1	1	1.2	1.1
vret	14989	36641	21552	22272	*2.3	1.0
Yb	721	590	794	527	1.2	1.5
zucA	6021	4560	2460	3114	1.3	1.2
